# An Extensive Study of Phenol Red Thread as a Novel Non-Invasive Tear Sampling Technique for Proteomics Studies: Comparison with Two Commonly Used Methods

**DOI:** 10.3390/ijms23158647

**Published:** 2022-08-03

**Authors:** Gábor Kecskeméti, Edit Tóth-Molnár, Tamás Janáky, Zoltán Szabó

**Affiliations:** 1 Department of Medical Chemistry, Albert Szent-Györgyi Medical School, University of Szeged, Dóm tér 8, H-6720 Szeged, Hungary; kecskemeti.gabor@med.u-szeged.hu (G.K.); janaky.tamas@med.u-szeged.hu (T.J.); 2 Department of Ophtalmology, Albert Szent-Györgyi Health Centre, University of Szeged, Korányi Fasor 10-11, H-6720 Szeged, Hungary; toth-molnar.edit@med.u-szeged.hu

**Keywords:** LC-MS, tear, proteomics, data independent analysis, mass spectrometry

## Abstract

Tear samples are considered in recent publications as easily, noninvasively collectible information sources for precision medicine. Their complex composition may aid the identification of biomarkers and the monitoring of the effectiveness of treatments for the eye and systemic diseases. Sample collection and processing are key steps in any analytical method, especially if subtle personal differences need to be detected. In this work, we evaluate the usability of a novel sample collection technique for human tear samples using phenol red threads (cotton thread treated with the pH indicator phenol red), which are efficiently used to measure tear volume in clinical diagnosis. The low invasiveness and low discomfort to the patients have already been demonstrated, but their applicability for proteomic sample collection has not yet been compared to other methods. We have shown, using various statistical approaches, the qualitative and quantitative differences in proteomic samples collected with this novel and two traditional methods using either glass capillaries or Schirmer’s paper strips. In all parameters studied, the phenol red threads proved to be equally or even more suitable than traditional methods. Based on detectability using different sampling methods, we have classified proteins in tear samples.

## 1. Introduction

The paradigm change from reactive to predictive, preventive, and personalized medicine requires novel and reliable methods that can provide a more precise diagnosis and patient stratification, detect early disease, elucidate the risk of disease, and predict disease outcome, response to therapy, and permit monitoring of therapeutic management. To this end, the discovery and validation of specific biomarkers/biomarker panels would be a promising approach. The source of biomarkers is crucial for the specificity, sensitivity, accuracy, and reliability of diagnostic tests and treatment targets [1]. Human tear fluid has attracted increasing interest in the last decades as a potential source of biomarkers of pathophysiological states, due to its accessibility, non-invasive nature of its sampling, moderate complexity, and responsiveness to ocular and systemic diseases [2]. Tear fluid contains proteins, such as enzymes, mucins, hormones, immunoglobulins, growth factors, neuropeptides, and cytokines along with lipids, salts, and carbohydrates [3]. This comprehensive biomolecule repertoire in human tears serves as a good source for biomarker discovery for diseases. The biggest advantage is that tears are proximal to the disease location (such as ocular surface disease, lacrimal gland disease, etc.) contrary to, e.g., cancer biomarkers in blood, where the related biomarker molecules could be distant from the source, and are highly diluted [4,5]. The tear fluid proteomic profile has been found to provide basic biological information for many ocular diseases, such as dry eye syndrome, blepharitis, keratoconus, thyroid eye disease, vernal keratoconjunctivitis, diabetic retinopathy, and primary open angle glaucoma [1,6]. Even though tear components are mainly derived from secretory glands, such as the lacrimal glands, the change of tear film composition is not only regulated by its secretion units. Protein molecules can enter the tear fluid through conjunctival blood vessels and, due to the overlap between the tear and plasma proteome, there may be opportunities to observe systemic responses in the tears. Several such examples thus far include breast cancer, type 2 diabetes, Alzheimer’s disease, multiple sclerosis, and rheumatoid arthritis [1,6]. Some systemic diseases may affect the eye so we can use ‘tears’ as a ‘window’ to assess systemic as well as ocular diseases.

The most frequently used tear sampling methods for proteomics analysis in both clinical and research settings involve the direct collection of tear fluid into a glass microcapillary tube or via an absorbent material such as Schirmer’s paper strips, threads, ophthalmic sponges, and polyester rods [7]. During capillary sampling, one end of the glass capillary is placed in the meniscus of the tear fluid and due to the capillary action, the tear flows from the conjunctival sac into the interior of the glass capillary. The Schirmer’s strip was originally a standard clinical tool used in many places to measure tear fluid volume, but it was found to be suitable for collecting tear samples too. Both sampling methods have advantages and disadvantages, they are often time demanding, uncomfortable for the patient, require medical professional assistance, and sometimes do not provide a sufficient amount of samples for analysis. We need a simple, fast, non-invasive, and reliable tear collection procedure that provides unbiased tear samples even from low-volume sampling (e.g., aqueous tear-deficient patients or experimental animals). Kurihashi et al. established a method of tear secretion measurement using fine cotton threads [8] which might meet the above requirements, and already proved to be an applicable sample collection method for analysis of small molecules [9].

The advancement in nano-scale liquid chromatography coupled MS (nanoLC-MS) that provides improved chromatographic separation of peptides, higher sensitivity, and extended dynamic ranges to identify > 1500 proteins, has opened up the possibility of tear biomarker research [6]. There are numerous studies and reviews in the literature on the investigation of protein profiles from differently collected tear samples using liquid chromatography coupled to mass spectrometry (LC-MS) [1,2,4,5,7]. It was shown that samples collected by Schirmer’s strip and capillary method have large quantitative and qualitative differences in their protein composition [6], therefore any new sample collection method should be evaluated in that respect too.

The goal of this study was to compare a new tear sampling procedure using phenol red thread (cotton thread treated with the pH indicator phenol red) (PRT) with the two most frequently used methods for sample collection in order to determine the protein profile of tears by liquid chromatography-mass spectrometry.

To the best of our knowledge, this is the first comprehensive study on the comparison of the commonly used Schirmer’s strip, microcapillary, and the novel PRT method in human tear proteomics analysis.

## 2. Results

### 2.1. The Choice of Sampling Method

Our goal was to introduce a reliable new sampling procedure, which provides us with a convenient way of collecting enough tear samples for quantitative proteomic studies. In this comparative study tears of the same 10 healthy donors were collected by three sampling methods and analyzed by the same proteomics procedure. General protocols for the use of glass capillary tubes (CAP) and Schirmer’s strips [10] were followed, and PRT as a new tear sampling device was included in this study. After sample collection, Schirmer’s strips were divided into a lower section (SL) which was in direct contact with the surface of the eyeball and eyelid, and the following 10 mm long upper section (SU) and processed separately.

A qualitative and quantitative proteomics study was performed on samples collected from the left and right eyes of all donors: altogether 20 PRT, 20 SU, 19 SL, and 18 CAP tear samples were analyzed. The total volume or protein content of all PRT, SU, and SL samples was satisfactory for our protocols; however, one SL sample was damaged during processing. In the case of two donors, the volume of tears collected with glass capillary tubes was insufficient (less than 1 µL) to determine the total protein content and perform proteomic sample preparation.

Both the volume and the total protein content of tears collected with the CAP sampling procedure showed large variance (7.9 ± 7.0 µL and 64.1 ± 44.9 µg, respectively). Tears are collected slowly and erratically, with interruptions due to blinking and eye movement, what—apart of individual variance—may explain the large fluctuation [4]. In contrast, the total protein content after the extraction of SL strips, 10 mm long SU strips, and whole PRT samples proved to be 42.0 ± 11.4 µg, 57.8 ± 15.5 µg, and 28.9 ± 11.2 µg, respectively.

### 2.2. Impact of MS Data Acquisition and Evaluation Method

Mass-spectrometry-based proteomics enables us to identify and quantify hundreds of thousands of proteins from different samples. However, the quality of the results is highly dependent on the experimental and computational workflows. A large number of proteomics software tools and algorithms have been published for data-independent acquisition (DIA) proteomics data processing. We applied DIA-NN software for spectral library creation, protein identification, and quantification, which is an integrated software suite, that exploits deep neural networks and new quantification and signal correction strategies [11]. Our DIA-NN workflow first was set up to use sample-specific spectral libraries generated by refining predicted libraries using six gas-phase-fractionated acquisitions (GPF-DIA) with 4 *m*/*z* precursor isolation windows from all four types of pooled samples. These sample-specific spectral libraries demonstrate the deepest proteome coverage of a given sample type. A combined spectral library was built by searching all four groups of GPF measurements in one against the same predicted spectral library. This combined library contains a total of 2583 proteins, any of which were present in a quantifiable amount in at least one pooled tear sample.

Data of DIA acquisitions collected from the analysis of individual samples (DIA-Q) were searched against both the sample-specific and combined libraries. The number of quantified peptides was 66.1 ± 42.9% higher when the search was carried out on the combined library. Searching against the combined library had a lower effect for SL samples than the PRT, SU, and CAP, as these samples contained the highest number of quantifiable peptides and contributed to the greatest extent to the size of the library.

Although the combined library originating from the GPF-DIA measurements from four pooled samples contain 2583 proteins, in the individual samples 1144 could be quantified with at least two peptides in the DIA-Q measurements.

### 2.3. Comparison of the Tear Proteomes of Different Sample Types

For the comparison of the proteomes detectable in our CAP, SL, SU, and PRT samples, the combined spectral library created from a GPF-DIA analysis of sample-specific pooled samples was chosen, as this demonstrates the deepest available protein coverage. All quantifiable proteins were included in this comparison, regardless of the number of peptides detected.

**Figure 1:**
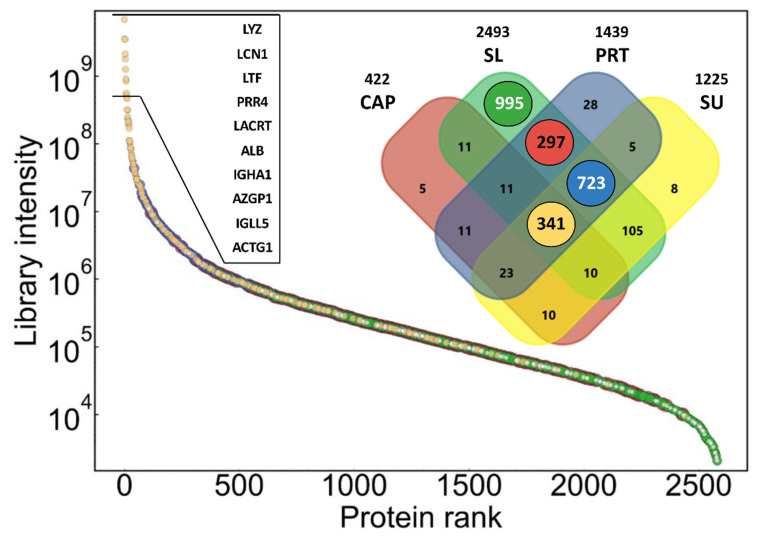
Summed intensity of proteins in combined spectral library as a function of protein intensity rank, demonstrating the dynamic range of identified proteins. Data points are colored according to highlighted subsets of the library as shown in the Venn diagram insert, to demonstrate contribution of different sample types to the library. Gene names of the top proteins are shown in the order of intensity rank.

The dynamic range and contribution of proteins in different types of samples to the combined library are shown in Figure 1. The library covers proteins with a range of more than six orders of magnitude in summed signal intensity. Number of proteins shown on the Venn diagram refers to number of protein groups, which may consist of more than one protein, and cannot be differentiated based on identified peptides. The lowest number of proteins could be identified in the pooled CAP sample (422 protein), while in the two indirect pooled tear samples (PRT and SU), a similar number of proteins could be detected (1439 and 1225) with a much higher overlap (1092 shared protein identifications). The SL samples were only included in the investigation to detect possible contaminants originating from the strong direct contact of the Schirmer’s strip with the ocular surface. This assumption is supported by the presence of nearly 1000 proteins, which were identified only in this type of pooled sample. These proteins are present in low abundance, according to their summed intensity (green dots in Figure 1). It must be noted that the parts of the PRTs in contact with the eye were not removed, which may explain the approximately 300 proteins shared by only PRT and SL samples (red dots in Figure 1). The majority of the most intense (10^6^–10^10^) proteins could be identified in all sample types, but there are many common proteins with lower intensity. Altogether, 341 protein groups could be detected in all the four sample types (ochre dots in Figure 1). The list and annotations of all proteins in the combined spectral library can be found in Table S1 in the Supplementary Materials.

**Figure 2:**
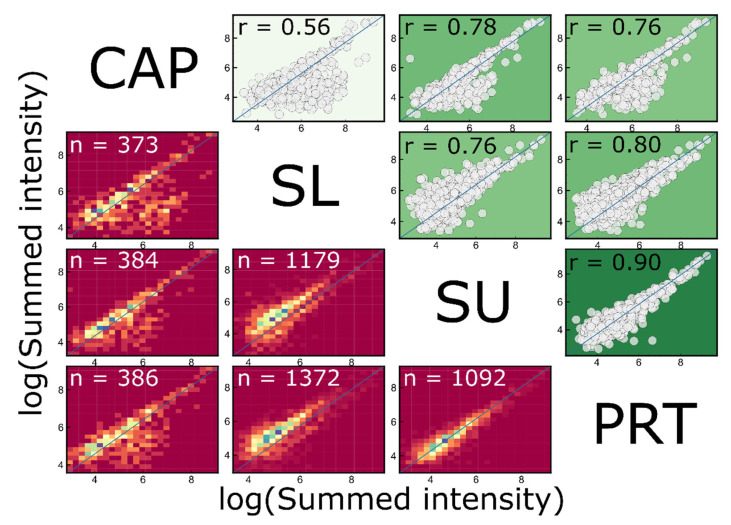
Correlation of intensities of proteins in different pooled samples. The depth of the background colors of scatter points on top-right is proportional to the value of actual Pearson correlation coefficients, which are shown on the 2D histograms at bottom-left. The number of the common sets of proteins (*n*) are given.

To study the quantitative similarity of different sample types, Pearson correlation coefficient values were calculated for the proteins quantified in sample type-specific pooled samples (Figure 2). This assay shows a strong correlation between PRT and SU samples (r = 0.90), but the pooled CAP sample also correlates satisfactorily with these samples (r = 0.76–0.78).

### 2.4. Clustering of Proteins in Different Types of Samples

The analysis of the spectral library created from the analysis of four different types of pooled samples in the previous section already highlighted the diversity of proteomes determined in samples collected by different methods, but the statistical classification of proteins based on sample-specific detectability is only possible on analysis of individual samples. It must be noted that the applied DIA approach, with match-between-run identifications enabled, can minimize the technical reasons for missing values, therefore this analysis reflects heterogeneity in sample composition.

**Figure 3:**
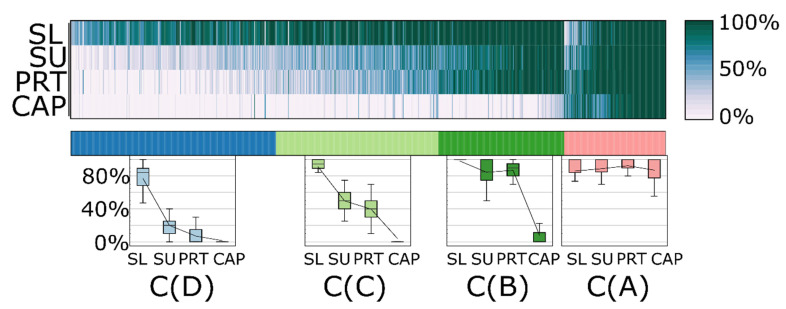
k-means (k = 4) cluster analysis of the quantified proteins based on the percentage of observations in samples of the different sampling procedures. Detection frequency of each protein is given as a heatmap on top. The boxplots on the bottom show the distributions of detectability of proteins in different sample types within each cluster.

A k-means cluster analysis based on sample-type specific detection frequency (in %)—was performed to classify the 1144 quantifiable proteins into four clusters (the number of clusters was predicted using the elbow method [12]) (Figure 3).

In Cluster A (pink), there were 195 proteins that could be measured with high frequency in all sample types (86–92% of the samples within any sample type). Cluster B (dark green) includes 242 proteins that were repeatedly measurable (84–98%) in samples from indirect procedures (SL, SU, and PRT), but they were quantifiable in only a few CAP samples (8%). The other two clusters consist of proteins that could be measured with high frequency in the SL samples, but in the SU and PRT samples only with medium (Cluster C), 312 proteins) or low (Cluster D), 395 proteins) frequency. Using this approach, proteins within the latest cluster may be marked as possible contaminants of tear samples. They have been found mainly in the SL samples, but rarely in any other sample group. On the other hand, proteins of Cluster A and B may be considered as common tear proteins, with the remark that CAP sampling provides reproducible detection for members of only Cluster A. It must be noted that PRT sampling produces the highest detection rate for proteins of those two clusters (see distributions on boxplots in Figure 3).

**Figure 4:**
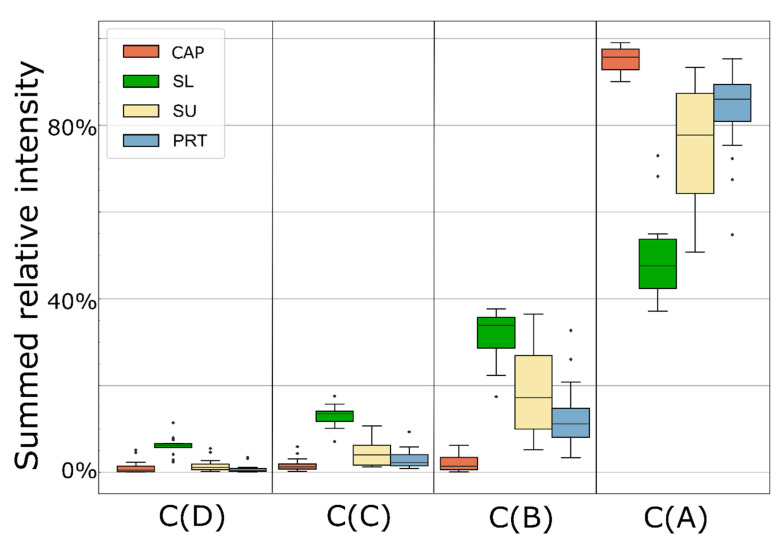
Boxplot of summed relative intensity of the four protein clusters in each sample type (n_CAP_ = 18, n_SL_ = 19, n_SU_ = 20, n_PRT_ = 20).

We have compared the summed relative intensities of the identified protein clusters in each type of sample (Figure 4). As expected, the highest level of the possible contaminating eye-surface proteins (395 and 317 proteins in Clusters D and C, respectively) were found in the SL samples, where on average 6.0% of the total intensity of all proteins is given by the proteins of Cluster D. CAP, SU, and PRT samples contain an average of 1.2, 1.5, and 0.7% proteins of that cluster. Proteins of Cluster C can be found at higher abundance in each sample type. Their values are still less than 5%, except for the SL samples (13% of total intensity). Proteins of Cluster B were detected only in a few CAP samples, and their intensity was low (on average 2%), while their relative abundance was highest in SL samples again (32% average). Members of Cluster A contribute the most to CAP sample protein intensity (95%), and slightly less than 50% in SL samples. The average abundance of these proteins is higher in PRT (83%) than in SU (76%) samples. For all clusters, the variance of PRT samples is lower than those in SU, but slightly higher than in CAP samples, which, however, have a less complex protein composition.

The list, quantitative data, annotations, and cluster assignments of all proteins quantified in the individual samples can be found in Table S2 in the Supplementary Materials.

### 2.5. Classification of Tear Proteins

Based on the detection frequency of proteins in samples collected using different sampling methods, we identified four clusters of proteins in the previous section. In order to give biological classification of the proteins in tear samples, each protein was annotated with available GO and UniProt terms. Fisher’s enrichment analysis was performed on these terms to identify common properties of proteins within each cluster. Here we discuss a few examples of the significantly enriched categories (Benjamini–Hochberg corrected FDR < 2%), which are different in different clusters and relevant to tear and tear sampling.

Proteins associated with specific intracellular localization GO terms, are enriched in Cluster D, which are specific to SL samples, e.g., 21% of Cluster D proteins are from mitochondrion, contrary to cluster A, which includes only 1% of such proteins. The general Cytoplasm GO subcellular localization is enriched in all the Clusters B, C, and D (40–71%), while in the Cluster A, which was the only cluster effectively sampled using capillaries, there were only 19% of such proteins.

Sixty-three percent of proteins in Cluster A are secreted, while only 9% of Cluster D proteins are annotated with this Uniprot Keyword. Eighty-five percent of the different Ig chains (36 of 40) detected in our samples are in Cluster A, which makes 17% of the proteins in this cluster. Most of the identified keratins (10 out of 12) frequently occur in all sample types, thus they are found in Cluster A.

**Figure 5:**
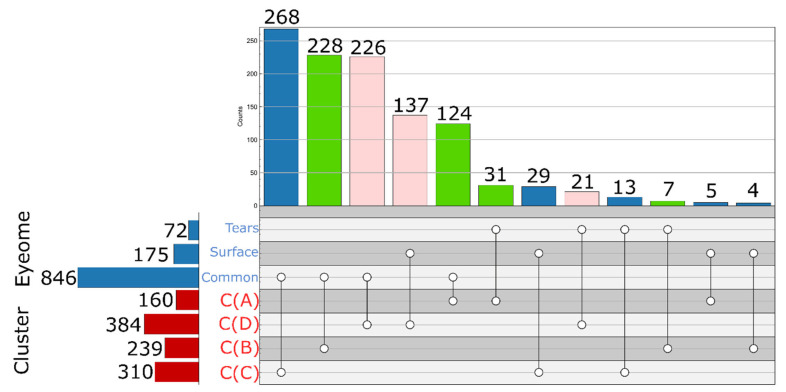
Count plot representation of the intersections of protein clusters and sections of the EyeOME [13] database. Clusters are those identified in Section 2.4. Proteins in the ‘Cornea’ or ’Sclera’ section of EyeOME were classified as Surface.

In addition to those general ontological annotations, some more eye-specific information was also added to clarify the origin of proteins in different clusters. The EyeOME [13] database collects a list of proteins identified in different parts of the human eye. For the classification of proteins in tear samples based on their possible origin, two groups were created: proteins which can be found in the ‘Tears’ section (including 1506 proteins) and an eye surface group from the ‘Cornea’ or ‘Sclera’ sections of the EyeOME database (1469 and 1895 proteins respectively). There is a large overlap in these assignments, 1213 proteins are common to tears and the eye surface in that database. Immunoglobulins are excluded from EyeOME, that is the main reason that not all, but 1093 proteins of the database were among the 1144 quantified proteins from our experiments, 846 of which are common to tears and the eye surface. Based on the overlap of clusters identified in Section 2.4 and the EyeOME assignments (shown in Figure 5), we can make further refinements of protein classification. The majority of the members of Cluster A are common (124), or specific to tears (31), thus we suggest classifying these as common tear fluid proteins. It must be noted that all Igs can be considered as such also, as those are found in Cluster A but excluded from the EyeOME. Most of the proteins of Cluster B (228 of 239) are common to tears and eye surface in EyeOME, rarely detected in CAP samples, therefore we can consider them as proteins of the lower layer of tear fluid and proteins easily and reproducibly collected from the eye surface using the indirect sampling methods. Altogether, these 437 proteins in Cluster A and B (392 in EyeOME) proteins we would classify as regular tear sample proteins, independent of origin (green in Figure 5). 

### 2.6. Intra- and Interpersonal Variances of Different Tear Samples

**Figure 6:**
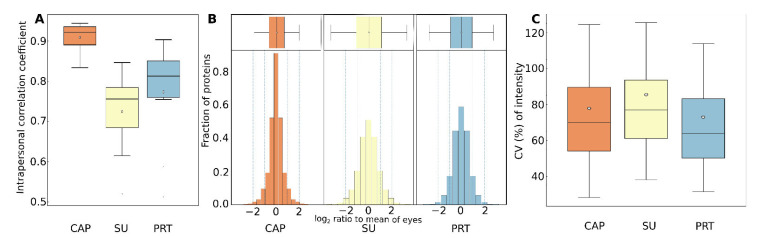
(**A**). Intrapersonal (left-to-right eye) Pearson correlation coefficients of samples of different sampling methods. (**B**). Distribution of protein log2 transformed intensity ratios measured in the two eyes relative to the average of the two eyes. (**C**). Distribution of coefficients of variation (CV%) for intensities of proteins in Cluster A in all samples of different sampling methods.

The differences in the composition of the tear samples collected from different persons, or from the two eyes of the same subject, may be a combination of several simultaneous reasons, including the effect of the sampling procedure on eye surface and tear secretion. In order to identify sampling induced effects, the protein composition of tear samples of the same person was compared using different methods, excluding the highly contaminated SL samples. Similar distributions of absolute protein abundance differences were observed in all sampling methods, however the Pearson correlation of intensities showed marked differences in the three tear fluid samples. The strongest intrapersonal correlation was found in the CAP samples (average of coefficients was 0.91, median 0.92), while PRT samples (average of 0.77, median 0.81), and SU samples (average of 0.73, median 0.76) presented weaker correlations. (Figure 6A). The ratio of the protein MS intensities measured in the two eyes relative to the average of the eyes was also calculated to represent the differences between the eyes. The log2 transformed distribution is the narrowest around 0 in the case of CAP samples, and widest is in Schirmer’s samples (Figure 6B). Eleven percent of data points has an absolute value higher than 1 (at least twofold difference relative to eye average) in the case of CAP samples, in Schirmer’s samples 21% and in PRT samples 14% has that high difference.

We calculated the overall variance (CV) of protein intensities in the whole sets of sample types. The CV distribution of the common 195 proteins of all sample types (Cluster A) are shown in Figure 6C. According to this, these proteins have a lower median CV in PRT samples (64%) than in either SU (77%) or CAP samples (70%).

### 2.7. Evaluation of the Proteomics Rotocol

The nanoLC-MS reproducibility was determined by triplicate injection of a randomly selected PRT sample. The number of identifications and the reproducibility of the relative intensities were compared at both protein and precursor levels; 3003 ± 21 precursors were quantified with the median CV of 0.17 in these measurements, corresponding to 548 ± 4 proteins with the median CV of 0.15. Based on these results, our procedure was suitable for the study of sample preparations and sampling procedures.

In order to evaluate the efficiency and reproducibility of protein extraction from PRT samples, three different extraction solutions (5% SDS, 100 mM ABC, and 1% acetic acid in water) were tested. Nine PRTs were soaked in a pooled capillary tear sample to ensure the same initial protein concentration and composition. Note that this experiment was limited by the number of proteins detectable from capillary samples, but comparison to the original pooled capillary sample provides satisfactory data on recovery and reproducibility for major tear proteins. There were no significant differences observed in the total amount of extracted proteins as determined by BCA assay (Table 1); however, the lowest reproducibility was found using acetic acid. The number of detected and quantified proteins, reproducibility (CV) of protein intensities and correlation to the original capillary sample were similar using the SDS and ABC protocols, but the acetic acid protocol performed worst regarding all these measures. 

Using either the SDS or ABC protocol, 84–86% of proteins can be recovered, and Pearson correlation with the original sample demonstrated, that the protein composition was not biased, the extract correctly demonstrates the original composition. Although ABC extraction seems to be suitable for protein extraction from PRT samples, the presence of a tenside (e.g., SDS) may help the extraction of the intracellular fraction that was excluded by our evaluation approach. Because of that, and for comparability with the protocol applied for Schirmer’s strips, we used the SDS approach in our analysis.

## 3. Discussion

The lacrimal apparatus supplies tear fluid to the surface of the eyeball and the eyelid, minimizing friction and cleaning the eye. Tear fluid and its components are actively secreted by various secretory units, including lacrimal glands, meibomian glands, accessory lacrimal glands, sebaceous glands of Zeis and Moll, and corneal and conjunctival cells. Additionally, the highly vascularized conjunctival tissue contributes with a large number of blood-derived compounds to the composition of tear fluid [14].

The main and accessory lacrimal glands secrete tear fluid into the lateral aspect of the superior conjunctival fornix, and the upper fornix. Then the fluid spread across the entire surface of the eye covering the anterior eyeball with a thin film, called preocular/precorneal tear film. The tear fluid flows from lateral to medial toward the tear drainage system at the inner canthus [15]. Tear production normally is about 0.5–2.2 μL/min, considering the total volume of 6–7 μL it causes 16% turnover per minute [4,16]. In addition to production, evaporation, absorption, and drainage are responsible for dynamic balance of the preocular tear film.

The preocular tear film is a thin fluid layer (2–6 μm) covering the ocular surface; it is the interface of the ocular surface with the environment. The precorneal tear film is now regarded as a complex blended two-layer structure comprising of a mucoaqueous gel layer lying beneath, but at least partly integrated with an overlying lipid layer [17].

Tears have been classified into three main types: basal, reflex, and psycho-emotional [18]. Basal tears, also known as non-stimulated tears, continuously coat the eye to keep it moist and protected. Reflex tear (or stimulated) is a higher lacrimal flow produced in response to external physical or chemical stimuli. Psycho-emotional tears are produced in response to joy, sadness, fear, and other emotional states. Each type of tears has many variants, and their differences are not always clear, as they sometimes overlap or are imprecise or controversial. It is more accurate to think of tear output as a continuum, whereby the rate of production is proportional to the degree of sensory or emotive stimulation [14]. The aqueous part of all types of tears are produced by the lacrimal glands and accessory lacrimal glands, but differ in their volume and composition [19].

As can be seen from the above, tear fluid is a dynamic mixture of substances with different origins and may have different composition depending on external physical or emotional factors. In addition, to the biological variance in tear composition, samples collected from the tear film may vary depending on the sampling position on the eye surface, depth of sampling of different fluid layers, changes induced by the sampling process (physical or emotional stimuli) and recovery from the sampling device.

Protein concentrations in normal tear fluid range from 6 to 11 mg/mL [5], but the protein concentration and composition of tear fluid samples are greatly influenced by the following factors [7,20]: (i) tear collection device (glass capillary tubes, Schirmer’s strips, threads, ophthalmic sponges, and polyester rods; (ii) types of collected tears (reflex, or non-stimulated tears), which might be affected by the tear sampling procedure, irritating stimuli like environmental fluctuations, physiological status or rubbing the skin with alcohol, anesthesia; (iii) location of tear sampling (the inferior temporal tear meniscus near the external canthus of the eyes, or the inferior conjunctival sac); (iv) whether sampling occurred from open or closed eye; (v) last, but not least, the sample processing (recovery from the sampling device) and the further analytical procedure and data analysis. As can be seen from the above, tear sampling method is definitely a major challenge and has the greatest significant influence on the precision and reproducibility of the analytical results.

Several tear sampling methods are available, and every sample collection method used must be assessed since it has a significant influence on the precision and reproducibility of the analytical results. Each sampling method has advantages and disadvantages; therefore, it is not easy to choose the appropriate one. The most often used tear sampling methods for proteomics analysis in both clinical and research settings involve direct collection of tear fluid into a glass microcapillary tube or via an absorbent material such as Schirmer’s paper strips, threads, ophthalmic sponges, and polyester rods. For determination of the protein profile of the tear sample the capillary tube and the Schirmer’s strip are used most frequently [7,20].

As both methods are routinely used for measuring tear volume in ophthalmology, and in many tears proteomics studies, much information has been gathered on their application. A comparison of the capillary, Schirmer’s strip and the new PRT tear collection methods is provided in Table 2. 

Collecting tear fluid by capillary tubes is generally found to be more convenient for patients, but because of fear of injury, some find it less desirable than the Schirmer’s strip. It can be time consuming, sometimes it can take up to 10 min to collect enough tears, because tears flow slowly and erratically, with interruptions due to blinking and eye movement [21]. Therefore, it less suitable for collecting samples from aqueous tear-deficient patients or animal models. To collect tears, capillary tube is usually placed close to the inferior temporal tear meniscus near the external canthus without touching the cornea, conjunctiva, and lower eyelid. If the sampling is performed by a specialist who has practice and experience in this collection method, then it does not induce reflex tearing, nor does it involve a potential risk of injury. However, the investigator has to hold the capillary tube for the duration of the sampling procedure, which entails constant and prolonged work on the open eye. Tear collection with capillary frequently requires previous stimulation or instillation of different volumes of saline into the cul-de-sac and collecting after sufficient mixing [7]. Capillary samples contains a higher percentage of proteins originating from extracellular region, protein containing complexes, and membrane [4,10,22]. In biological processes, immune response, complement pathway, and tissue development proteins dominate more frequently in capillary samples. 

Schirmer’s test is well-established in clinical ophthalmic practice to measure tear secretion [23,24]. Tear collection is performed using a special filter paper strip (5 mm wide and 35 mm long) with the bent end placed between the palpebral conjunctiva of the lower eyelid and the bulbar conjunctiva of the eye. The eye is then closed for 5 min while the tear fluid absorbs into the filter paper. The test can be performed with or without the use of anesthetics. Although Schirmer’s strips have been considered a convenient and easy to perform method of tear collection, their use can cause strong irritation, leading to reflex tearing that results in larger but more diluted samples. The strip is in contact with the highly vascularized conjunctiva and can injure its surface and microvasculature. This damage and induction of the secretion of reflex tears by mechanical irritation likely distort the protein-profile of tear samples. Due to the trauma, these kinds of samples contain proteins not only from tears but also from surrounding tissues and blood [4,25]. An increased number of cell and organelle-specific (intracellular) proteins has been reported that contaminate tear samples [10].

Collection of tears with Schirmer’s strip is an indirect method carried out using absorbing supports. Proteomics analysis of these kinds of samples requires either centrifugation of tear fluid or extraction of proteins (before digestion) or peptides (after in-strip digestion) from the paper strips. A disadvantage of this absorbent-based sampling method is that different extraction procedures may result in varying protein profiles. It was recently reported [36] that the elution of proteins from Schirmer’s strips varies significantly between different brands of the filter paper because of their distinct absorptive properties. Apart from the variety of clinical procedures used to perform Schirmer’s test, it seems likely that one of the causes of the variability of Schirmer’s test between studies is related to the use of different Schirmer’s test strips. Unfortunately, there is no standardization of commercial strips, even though the need for standardization was recognized over fifty years ago. The maximum volume of absorption on 10 mm long piece of Schirmer’s strip was found to be around 9 µL [27], but it may also depend on strip material.

Phenol red thread, like the Schirmer’s test, is a widespread clinical test for measuring tear volume [28,32]. In the detection of dry eye, PRT is equally sensitive, but it has many advantages over Schirmer’s test. Sampling can be performed in a significantly shorter time (15 s vs. 5 min), which is much more convenient for patients. It can also be performed on children [11] and it has a significantly smaller contact area causing minimal irritation and minimal induction of reflex tearing [37]. It has the advantage that it is not only suitable for human tear sampling but has been shown to better evaluate tear secretion in small animals with lower tear volume such as birds [38] and rodents [39]. Phenol red thread collects around 3 μL of tear fluid [21], and this volume is low compared to an average basal tear volume. The inability of PRT to absorb the entire volume of basal tears means that the collected initial sample is almost pure basal tears; however, it can be used for the collection of reflex tears if applied after stimulus [21], which can be a big advantage over other tear collection methods.

In our experiments, both absorbent-based approaches (SU and PRT samples) allowed the collection of low-volume samples, and the amounts of proteins were sufficient to perform several proteomic analyses even from those two donors whose CAP tear samples were not sufficient. The safe application of capillary approach without touching the eye surface, requires larger tear volume with a thick tear film. This makes it less suitable for the collection of tear samples from aqueous tear-deficient patients [22,29], but there is no such limitation with the indirect methods, where the soft sampling material is immersed in the tear film.

The observable proteome with the two most frequently used tear fluid sampling methods have been compared several times; most of these studies conclude that although both methods can be used, the capillary and Schirmer’s strip tear collection methods still result in different protein compositions [7,10,22,23]. It was assumed that this is because Schirmer’s strip results in an increased tear production due to possible irritation and contains proteins not only from tear fluid but also from tissues via direct eye contact [34].

Our findings are consistent with these results, as more proteins were identified in the samples collected with the Schirmer’s strips compared to the capillary samples. The SL samples contain an even larger number of proteins than SU samples being in direct contact with the ocular surface. The number of identified proteins in the novel PRT samples was similarly high (1439), as in the SU samples, and protein intensities showed a strong correlation with other sample types. This proves the applicability of the PRT method to efficiently collect samples for proteomics LC-MS analysis with a composition comparable to samples from other methods.

Ma et al. recently summarized [6] proteomics datasets from tear films using either capillary or Schirmer’s strips by different research groups. They collected 1892 proteins from 11 publications and found 435 proteins common to capillary and Schirmer’s strip samples. Based on gene names, we matched those proteins to our dataset, to evaluate the overlap of our data with those recent results. Of all those proteins, 1656 were identified in our experiments, most of them in SL samples (1623), while 1238, 1089, and 382 proteins were found in the PRT, SU, and CAP samples, respectively. Regarding the 435 proteins designated as common by Ma and coworkers [6], we could identify 432 in total, 426, 406, 392, and 192 in SL, PRT, SU, and CAP samples, respectively. These results highlight the comparable proteomics usability of PRT not just with SU samples processed and analyzed in our laboratory, but with numerous other methods.

Akkurt Arslan et al. [40] processed their Schirmer’s samples in a similar manner and found that 1153 proteins were identifiable in their SL samples, while 1107 proteins were identified in their SU samples. The significantly larger number of proteins identified in our SL samples may be explained by the MS method (DIA in our case vs. DDA) [6] and by the larger number of samples (20 SL samples from 10 individuals in our study vs. four SL samples from two individuals). Contamination occurs randomly, thus increasing the number of samples increases the chance of identification. It must be noted that only pooled samples were analyzed in that study and no quantitative comparisons were made, so no information is available on identification repeatability and quantitative variability.

The identification of such a large number of proteins in our experiments was made possible by the application of the GPF-DIA LC-MS method. By the application of a combined spectral library, it was possible to quantify a higher number of useful proteins present in the tear fluid under normal conditions in a single nanoLC-MS run. A similar result was observed by Nättinen et al. [10], that almost twice as much protein could be quantified in tear samples collected by capillary using a combined spectral library instead of capillary type-specific one. They also did not find increased number of proteins in Schirmer’s type samples searched in a combined library. Green-Church et al. [22] have previously demonstrated that although the proteins detected in capillary samples were mainly extracellular, tear samples collected by Schirmer’s strip contained a large number of additional cellular proteins. Using a combined spectrum library, those proteins that could not be detected with the sample-specific libraries became easier to identify. Thus, it can be used as a quality control to identify tear samples that contain higher levels of contaminating proteins.

Based on detection frequency in all sample types, we could identify four clusters of proteins, and by comparing of these clusters to the EyeOME dataset we identified 437 proteins (Cluster A + B) which can be considered as common tear fluid proteins, but only 155 of those (in addition to immunoglobulins) can be effectively sampled by our capillary protocol. PRT has however has little higher efficiency in sampling of all those proteins than the Schirmer’s strip.

The summed relative intensity of the possible contaminant proteins originating from the eye surface (Cluster D) is the lowest in the PRT samples (less than 1%). This is very interesting because the entire thread was processed; the part in direct contact with the surface of the eyeball and the eyelids was not removed, unlike in the case of SU samples. This may be a consequence of the smaller diameter and the smaller and smoother surface of PRT fibers compared with Schirmer’s paper strips.

According to the Gene Ontology analysis of protein clusters, we can conclude that intracellular proteins originating from the eye surface and lower layers of the tear film are increasing the size of the proteome sampled by the indirect methods compared to capillary. These proteins are most effectively collected on the surface of the lower part of the Schirmer’s strip which is in direct contact with the surface of the eyeball and eyelids. Therefore, we conclude that those proteins may be designated as contaminants, if study of tear fluid is the goal, but may provide diagnostic information on eye surface, if specific sample collection methods can be applied for their reproducible analysis.

We can compare our clustering with the dataset of Ma et al. [6] described above, in which they found 435 proteins common in the literature data to Schirmer’s strip and capillary samples. We could quantify 387 proteins of those, 68% of which can be found in Cluster A and B, while only 11% are in Cluster D. This shows high similarity between the classifications, despite the different approaches. They concluded that those common proteins are present at high abundance regardless of sample collection, which we also demonstrated (Figure 4). Our observation, that the rarely identified, low intensity proteins can be found with high frequency and at higher intensity in the SL samples, confirms the ocular surface origin of those proteins.

In order to validate the application of PRT in tear biomarker analysis, we collected tear biomarkers from recent reviews of literature data [6,41,42,43,44,45]. We identified 87 proteins in our dataset that were previously assigned as putative biomarkers, 90% of which (78 proteins) are among the proteins which were commonly detected by the indirect sampling methods. Considering these results, PRT is a suitable sampling method for the studying biomarkers of both eye-specific and systemic diseases.

Sample processing is one of the most crucial processes in proteomics research; this pre-analytical step can bias both protein composition and quantification. Absorbent-based tear collection requires a pre-analytical step to elute/extract proteins from a paper strip, sponge, thread, etc. Several extraction conditions were proposed, by varying extraction solvent, volume, agitation time, and temperature to recover proteins from Schirmer’s strips [4,35] and small bioactive molecules from PRT [21].

Tear proteins absorb on PRT by varying strengths of intermolecular interactions with thread cellulose. The small surface and volume of PRT compared to a Schirmer’s strip results in less interactions between proteins and thread. This can make it easier to elute/extract proteins from PRT. Our results supporting this theory because using either the SDS or ABC protocol, 84–86% of proteins can be recovered, and protein composition was not biased, the extract correctly demonstrated the original composition with excellent reproducibility. Finally, phenol red thread is produced only by one company (Showa Yakuhin Kako Co., Ltd., Tokyo, Japan), so using the same tear collection device in different clinical and laboratory studies would reduce potential variables in tear analysis.

We have also evaluated the effect of sampling on variance of results. We have found a stronger correlation and smaller differences between samples from the two eyes of the same person using the PRT method compared to the Schirmer’s test (SU samples). The interpersonal protein intensity variances within all the healthy subjects were the lowest in the PRT samples (median of 64% for the common the proteins), considerably lower than in the SU samples (median of 77%).

These lower intra- and interpersonal variances may be attributed to the lower induction of reflex tear formation during sampling compared to Schirmer’s strip [28,37]. At the same time, the lower volume quickly collected by PRT ensures the collection of reproducible pure basal tear [21], thus making it more suitable for comparative analysis.

## 4. Materials and Methods

### 4.1. Materials/Reagents

Calibrated glass microcapillary tubes (20 mL) were manufactured by Drummond Scientific Company (Broomall, PA, USA), Schirmer’s strips (I-Dew Tearstrips) by Entod Research Cell UK Ltd. (London, UK) and PRT (Zone-Quick test) by Showa Yakuhin Kako Co., Ltd. (Tokyo, Japan).

Reagents, such as ammonium bicarbonate (ABC), dithiothreitol (DTT), iodoacetamide (IAA), and sodium dodecyl sulfate (SDS) were purchased from Sigma-Aldrich (Darmstadt, Germany), acetone from Merck (Darmstadt, Germany), trypsin, and formic acid (FA) from Thermo Scientific (Rockford, IL, USA). Water, acetonitrile, and acetic acid were delivered by VWR (Debrecen, Hungary).

### 4.2. Samples

Five healthy female and five healthy male volunteers participated in the experiment, aged between 20 and 33 years. All subjects included in this study were enrolled at the Department of Ophthalmology, University of Szeged. Approval for the human study was granted by the local Ethical Committee of the University of Szeged (108/2019-SZTE), and the study protocol adhered to the tenets of the most recent revision of the Declaration of Helsinki for experiments involving human subjects. All subjects enrolled in this study provided voluntary written informed consent.

For each subject, tear fluid samples were collected from both right and left eyes using three different sampling techniques during the same morning visit. More specifically, tear samples were collected (in the order of sampling) using glass capillaries (CAP), PRT, and Schirmer’s strips. For CAP sampling glass microcapillary tubes were introduced into the ventral cul-de-sac of the conjunctiva of the opened eyes for an average of 2 min without contact to the cornea, conjunctiva, and lower eyelid. PRTs and Schirmer’s strips were placed over the lid margin at the junction of the lateral and middle thirds of the lower eyelids. The sampling lasted 15 s for PRTs and 5 min for Schirmer’s strips while subjects closed their eyes without an anesthetic. In order to obtain tear samples from the resting eyes, there was a 30-min break between the different sample collection methods. Samples were immediately placed into sterile plastic microtubes and frozen at −80 °C until the analytical procedure. During sample preparation, the Schirmer’s strips were divided into lower (SL) and upper (SU) parts by cutting strips at the zero line and at the sign of 10 mm, respectively. The SL represent the rounded portions of the paper that were in direct contact with the eye surface, while the SU samples are the subsequent 10 mm sections. The rest of the strips were not processed.

### 4.3. Protein Extraction

All the SL, SU, and PRT samples were extracted individually by adding 100 µL 5% SDS in ABC (pH 8.0, 100 mM) containing 0.25% protease inhibitor cocktail (Sigma-Aldrich, Rockford, IL, USA). The samples were vortexed and incubated in a thermal shaker at room temperature for 1 h at 350 RPM. After a centrifugation at 12,000× *g*, 10 min, 4 °C, the supernatants of the samples were transferred into new tubes. 

The capillary tear samples were diluted using ABC (pH 8.0, 100 mM) containing 5% SDS and 0.25% protease inhibitor cocktail before determining the protein concentration.

### 4.4. Evaluation of Protein Extraction Methods from PRT

To examine the protein extraction efficiency from PRTs, different extraction solvents were tested. A pooled tear sample was collected in a low protein binding tube from three healthy persons using glass capillaries. Nine pieces of 5 cm long PRT threads were inserted into the pooled tear sample for 15 sec. After that the treads were divided into three groups. The protein contents of the threads were extracted using 100 µL of (1) 5% SDS in ABC (pH 8.0, 100 mM), (2) ABC (pH 8.0, 100 mM), (3) 1% acetic acid in water. All extraction liquids contained 0.25% protease inhibitor cocktail and were tested on 3 replicates. The samples were incubated in a thermal shaker at room temperature for 1 h at 350 RPM. After a centrifugation at 12,000× *g*, 10 min, 4 °C, the supernatants of the samples were transferred into new tubes.

### 4.5. Sample Preparation

The protein contents of all samples were determined using BCA Protein Assay (Thermo Scientific, Rockford, IL, USA) according to the manufacturer’s protocol. Samples containing 10 µg protein were processed using an on-pellet digestion protocol [46] with slight modifications. Briefly, the samples were reduced with 10 mM DTT at 60 °C for 30 min and alkylated with 20 mM IAA in dark at room temperature for 30 min. The protein content was precipitated by adding a 7-fold volume of ice-cold acetone and incubated at −20 °C overnight. After centrifugation with 15,000× *g*, 10 min, 4 °C the supernatant was discarded. The protein pellet was washed twice with 500 µL acetone/water (85:15, *v*/*v*) mixture. After centrifugation with 14,000× *g*, 10 min, 4 °C, the protein pellet was dissolved in 15 µL RapiGest SF Surfactant (Waters, Milford, MA, USA) and was incubated at 100 °C for 5 min. After being cooled to room temperature, 65 µL ABC (pH 8.0, 100 mM) and 0.25 µg trypsin in 5 µL ABC (pH 8.0, 100 mM) were added to the mixtures. The samples were incubated at 37 °C for 30 min and another 0.25 µg trypsin in 5 µL ABC (pH 8.0, 100 mM) was added and the mixture was digested at 37 °C for 5.5 h. Digestion was stopped by the addition of 1 µL concentrated formic acid. The samples were centrifuged with 12,000× *g*, 10 min, 4 °C, and 2 µL of the supernatant was injected to the nanoLC-MS system.

### 4.6. NanoLC-MS Measurements

NanoLC-MS/MS analysis was carried out on a Waters ACQUITY UPLC M-Class LC system (Waters, Milford, MA, USA) coupled with a Q Exactive^TM^ Plus Hybrid Quadrupole-Orbitrap^TM^ mass spectrometer (Thermo Fisher Scientific, Waltham, MA, USA). Symmetry^®^ C18 (100 Å, 5 µm, 180 µm × 20 mm) trap column was used for trapping and desalting the samples. Chromatographic separation of peptides was accomplished on an ACQUITY UPLC^®^ M-Class Peptide BEH C18 analytical column (130 Å, 1.7 µm, 75 µm × 250 mm) at 45 °C by gradient elution (linear gradient from 3% solvent B to 40% solvent B in 70 min, followed by a 30 min washing and equilibrating gradient). Water (solvent A) and acetonitrile (solvent B), both containing 0.1% formic acid were used as mobile phases at a flow rate of 350 nL/min. The sample temperature was maintained at 5 °C. The mass spectrometer was operated using the equipped Nanospray Flex Ion Source.

Data acquisition was performed using XcaliburTM 4.1 (Thermo Fisher Scientific, Waltham, MA, USA). 

### 4.7. DIA Acquisition and Data Processing

Data for creating sample-specific (CAP, SL, SU, PRT) spectrum libraries were collected from LC-MS analysis of four sample-group-specific pooled samples. The mass spectrometer was configured to acquire six gas-phase fractionated (DIA-GPF) acquisitions with 4 *m*/*z* precursor isolation windows at 17,500 resolution to hit an AGC target 1 × 10^6^ with a maximum inject time of 120 ms. In the GPF-DIA measurements, an overlapping window pattern was adjusted from the previously optimized mass ranges (395–505, 495–605, 595–705, 695–805, 795–905, and 895–1005 *m*/*z*). 

For the quantitative analysis of individual samples, a single LC-MS run with DIA acquisitions (DIA-Q) from 395 to 1020 *m*/*z* was used with 27 × 22 *m*/*z* overlapping precursor isolation windows at 17,500 resolution to hit an AGC target 2 × 10^5^ and the maximum inject time was set to auto.

DIA-NN 1.8 software [47] was applied for database search. First, a predicted spectral library was generated from the Uniprot Human Reference FASTA proteome (20,575 genes, one protein per gene). The experimental sample-specific libraries were created by searching the GPF measurements of each sample groups separately against the predicted spectral library. The experimental combined spectral library was built by searching all the four groups of GPF measurements in one against the same predicted spectral library. All the individual samples were searched against both the experimental sample-specific and combined spectral libraries to detect all peptides and proteins that can be quantified. DIA-NN was configured with the default settings with the following modifications: the maximum number of missed cleavages and variable modifications were set to 2, methionine oxidation was added, and precursor *m*/*z* range was adjusted to 380–1020. Search results were filtered for 1% FDR at the level of unique genes. Further data processing, statistical analysis and creation of figures were carried out in Perseus (version 1.6.15.0) [48] and Instantclue (version 0.1.1) [49] software. In all quantitative statistical analysis, only proteins identified with at least two unique peptides were included. Because of the large biological variance among samples, for better comparability, not the raw intensities of individual proteins but their relative proportion in the samples were used. Therefore, intensities of the proteins were divided by the sum of intensities of all proteins as normalization and log2 transformation was performed on all datasets. 

The analyzed proteins were annotated from Gene Ontology [50] and UniProt (https://www.uniprot.org) (accessed on 12 April 2022)

## 5. Conclusions

A crucial starting point for biomarker discovery in the tear proteome is the in-depth investigation of tear proteins in healthy subjects. Thanks to recent methodological advances, MS-based tear proteomics can guarantee the high accuracy and sensitivity necessary for biomarker discovery and single-tear analysis. To determine how the tear fluid sampling method affects the protein profiles obtained, tear samples from healthy persons, collected by using glass capillary, Schirmer’s strip and phenol red threads, were analyzed and statistically evaluated. Compared to the two widely used tear collection methods, the PRT was proven to be a fast, reproducible, and reliable sampling procedure, providing tears from patients whose capillary samples were not enough for proteomics analysis. The tear sampling method substantially impacted the proteomic profiles of individual samples determined by nanoLC-MS/MS. The number of proteins considered to be “tear proteins” was higher in the Schirmer’s strip and PRT; however, samples collected by PRT contain even lower amounts of “contaminant” proteins than the eye surface. According to our experience, PRT can be used successfully for proteomic analysis not only of human but also of mouse tears (data not shown). 

Unique properties of PRT allow fast, non-invasive collection of small amounts of tears and gives almost pure basal tear samples with unbiased protein composition. These characteristics make PRT sampling methods ideal for proteomics analysis of tear fluid and the discovery of protein biomarkers of ocular and systemic diseases, therefore in such studies it could replace current methods of sample collection.

The mass spectrometric data acquisition and evaluation method also influence the proteomics results. Creating and using a combined instead of sample-specific spectrum libraries resulted in a higher number of quantified proteins in each sample. Application of this method not only increased the number of useful proteins but also helped to identify and quantify different clusters of proteins, based on detectability using different sampling techniques. This may help future studies to focus on the most informative sub-proteomes of the human tears and eye surface.

## Figures and Tables

**Table 1:** Comparison of tear protein extraction methods from PRT.

	Total Amount of Proteins (µg)	Number of Quantified Proteins	Percentage of the Total Number of Proteins	Median CV of Intensity	Pearson Correlation to Capillary (r)
Capillary	-	236	100.0	0.17	-
SDS	41.5 ± 3.3	198	83.9	0.19	0.941
ABC	36.4 ± 1.16	203	86.0	0.15	0.946
Acetic acid	40.9 ± 6.78	152	64.4	0.23	0.851

**Table 2:** Characteristics of tear collection methods for proteomics analysis.

Parameter	Glass Capillary (CAP)	Schirmer’s Strip (SU)	PRT
Average protein amount recovered *	60 µg	58 µg	29 µg
Number of proteins identified *	422	1225	1439
Average intracellular “contaminant” protein MS intensity *	1.2%	1.5%	0.7%
Median CV of major proteins *	70%	77%	64%
Tear volume collected	3–10 µL [26]	~9 µL/cm [27]	~3–4 µL [21]
Tear collection time	Up to 5 min [20]	5 min [28]	15–20 s [28]
Contact with eye	No/minimal [22,29]	Strong contact with cornea, conjunctiva and lower eye-lid [7,29,30]	Mild contact with cornea, conjunctiva and lower eye-lid
Site of tear sampling	Variable [7,20]	Variable [7,20]	Standard [9,20,28]
Diagnostic repeatability (volume)		Poor ** [28,31]	Good [32]
Open/closed eye	Open	Open/Closed	Open/Closed [33]
Invasiveness	Low [34]	High [34]	Low/Medium
Discomfort (subjective)	No [23]	Yes [28]	No [28,32]
Sensitivity in detecting dry eye disease	Low	Low [28]	High [32]
Induces reflex tearing with higher flow rate	No [7]	Yes [28,34]	Minimally [28]
Sample processing	-	Cutting and protein extraction or centrifugation [2,35]	Protein extraction *
Sampling device material/source	Variable	Variable [36]	Uniform [20]
Risk of injury	Yes	No [23]	No
Cellular/plasma contamination	Low [10,22]	High [4,10,22,29,30]	Low *
Tear collection requires specialist	Yes [23]	No	No
Continuous intervention during sample collection	Yes [23]	No [23]	No

* Data from current work, ** Can be improved with anesthesia (Type II test) [26].

## Data Availability

The combined spectral library and quantitative results from DIA-NN analysis are available through Zenodo at: https://doi.org/10.5281/zenodo.6757957 (accessed on 24 June 2022).

## Supplementary Materials

The following supporting information can be downloaded at: https://www.mdpi.com/article/10.3390/ijms23158647/s1.

## Abbreviations

ABC	ammonium bicarbonate
AGC	Automatic gain control
CAP	capillary
CV	coefficient of variation
DDA	data dependent acquisition
DIA	data independent acquisition
DTT	dithiothreitol
EyeOME	The Human Eye Proteome Project
FA	formic acid
FDR	false discovery rate
HCD	Higher-energy collisional dissociation
IAA	iodoacetamide
LC/MS	Liquid chromatography–mass spectrometry
m/z	mass/charge
M	mol/L
MS/MS	tandem mass spectrometry
PRT	phenol red thread
SDS	sodium dodecyl sulfate
SL	Schirmer’s strip lower part
SU	Schirmer’s strip upper part
